# Association of air purifier usage during pregnancy with adverse birth outcomes: the Japan Environment and Children’s Study

**DOI:** 10.1186/s12889-024-20802-4

**Published:** 2024-12-18

**Authors:** Hidekuni Inadera, Kenta Matsumura, Haruka Kasamatsu, Kanako Shimada, Akiko Kitase, Akiko Tsuchida, the Japan Environment and Children’s Study Group, Hidekuni Inadera, Michihiro Kamijima, Shin Yamazaki, Yukihiro Ohya, Reiko Kishi, Nobuo Yaegashi, Koichi Hashimoto, Shuichi Ito, Zentaro Yamagata, Takeo Nakayama, Tomotaka Sobue, Masayuki Shima, Seiji Kageyama, Narufumi Suganuma, Shoichi Ohga, Takahiko Katoh

**Affiliations:** 1 Department of Public Health, Faculty of Medicine, University of Toyama, Toyama, Japan; 2 Toyama Regional Center for JECS, University of Toyama, Toyama, Japan; 3 National Institute for Environmental Studies, Ibaraki, Japan

**Keywords:** Air Purifier, Birth cohort, Low birth weight, Preterm birth, Small for gestational age

## Abstract

**Objective:**

Previous studies have reported that ambient air pollutants such as PM2.5 can increase the risk of adverse birth outcomes. The objective of this study was to ascertain whether air purifier usage during pregnancy is associated with a lower risk of adverse birth outcomes in a large Japanese birth cohort.

**Methods:**

We conducted a prospective cohort analysis using data from the Japan Environment and Children’s Study. Use of air purifiers during pregnancy was assessed using a self-administered questionnaire. Primary outcomes were the prevalence of preterm birth (PTB), small for gestational age (SGA), and low birth weight (LBW). Logistic regression analysis was performed to estimate odds ratios (ORs) and 95% confidence intervals (CIs).

**Results:**

The prevalence of outcomes was 4.5% for PTB, 7.4% for SGA, and 8.1% for LBW. The crude model analysis revealed that PTB, SGA, and LBW showed lower ORs in the group that used an air purifier, although the association disappeared in the adjusted model except for SGA (OR: 0.94; 95% CI: 0.89, 1.00, *p* = 0.048) and LBW (OR: 0.93; 95% CI: 0.88, 0.98, *p* = 0.003). Subgroup analysis stratified by infant sex revealed that the lower OR for LBW was observed only in male infants.

**Conclusions:**

Our results suggest that avoiding maternal air pollution exposure during pregnancy may be useful in preventing adverse birth outcomes. These findings provide evidence supporting the development of protective measures against air pollutants in the gestational period by relevant health agencies.

**Supplementary Information:**

The online version contains supplementary material available at 10.1186/s12889-024-20802-4.

## Background

Adverse birth outcomes (ABOs) are a contributing factor to a number of social and economic burdens worldwide, making them a major public health concern. ABOs include preterm birth (PTB), small for gestational age (SGA), and low birth weight (LBW). PTB, which is defined as less than 37 weeks’ gestation, is associated with adverse outcomes for both maternal and child health [1]. Premature infants may also have long-term impairment and experience social inequality in terms of their health outcomes in adulthood [2]. SGA is defined as having a birth weight below the 10th percentile and is a predictor of poor fetal growth [3]. LBW, which is defined as birth weight (BW) less than 2500 g, is associated with not only perinatal morbidity and mortality but also other cardio-metabolic disorders later in life [4]. Given that ABOs can have lifelong consequences, it is important to clarify the influencing factors and to identify effective interventions or potential countermeasures.

Although the causes of ABOs remain to be clarified, one possibility is intrauterine exposure to air pollutants. Fine particulate matter has attracted special attention because of its unique characteristics, including small diameter, large surface area, long suspension time in the air, and toxic effects on the human body [5]. Air pollutants are hypothesized to cross the placental barrier, disrupting fetal–maternal circulation and affecting fetal growth. For example, fine particulate matter with an aerodynamic diameter of 2.5 μm or less (PM2.5) can enter the blood circulation, pass through the placental barrier, and trigger the disturbance of homeostasis *in utero*, causing abnormal fetal development [6, 7]. PM2.5 can cause systemic inflammation, oxidative stress, and endothelial dysfunction, leading to ABOs [8, 9]. Indeed, observational studies have reported an association of perinatal exposure to PM2.5 with ABOs, including PTB, SGA, and LBW [10, 11]. Interestingly, the Chinese government successfully reduced pollutant concentrations in the air during the 2008 Summer Beijing Olympics and Paralympics and there was a subsequent increase in BW [12]. Another natural experiment revealed a decreased risk of PTB in women who were pregnant following the closure of a local steel mill compared with those who were pregnant while it was still operating [13]. Taken together, these findings suggest that reducing maternal exposure to air pollutants during pregnancy can reduce the prevalence of ABOs.

In Japan, pregnant women tend to spend more time at home and are thus exposed to pollutants from indoor air for longer durations [14]. One previous study reported that women who were exposed to high levels of indoor air pollution from solid fuel use delivered infants whose BW was, on average, 96.6 g lower [15]. Recent studies have also indicated that indoor air pollution resulting from solid fuel use for cooking or heating, paternal smoking, and new furniture was associated with ABOs [16, 17]. One possible countermeasure to avoid indoor air pollution is the use of air purifiers in the home. Air purifiers target both outdoor pollution that infiltrates the indoor environment and indoor-generated pollution from cigarettes, cooking, heating, and other sources. Air purifiers can effectively reduce these indoor air pollutants [18, 19]. We have previously reported an association between maternal air purifier use during pregnancy and decreased prevalence of neurodevelopmental delay in infants [20, 21]. We speculate that air purifier use can be an effective countermeasure by reducing air pollution in the home, but the relationship between such use and the prevalence of ABOs remains to be clarified.

Using a large dataset from the Japan Environment and Children’s Study (JECS), in this study we prospectively examined the hypothesis that the use of air purifiers to reduce airborne pollutants during pregnancy would be associated with a reduced risk of ABOs.

## Methods

### Study design

The Japan Environment and Children’s Study (JECS) is a government-funded, nationwide birth cohort study that investigates the impact of environmental factors on children’s health and development. In total, 103,057 pregnancies were registered at 15 regional centers across Japan between January 2011 and March 2014. Detailed descriptions of the JECS and baseline characteristics have been published elsewhere [22–24]. In brief, mothers were recruited during pregnancy. Written informed consent was obtained from all participants following a face-to-face explanation of the study. All procedures contributing to the present work comply with the principles of the 1975 Declaration of Helsinki, as revised in 2008. The JECS protocol was reviewed and approved by the Ministry of the Environment’s Institutional Review Board on Epidemiological Studies (100910001) and the ethics committees of all participating institutions. This specific study was also approved by the Institutional Review Board of the University of Toyama (R2023221).

### Study data

**Fig. 1:**
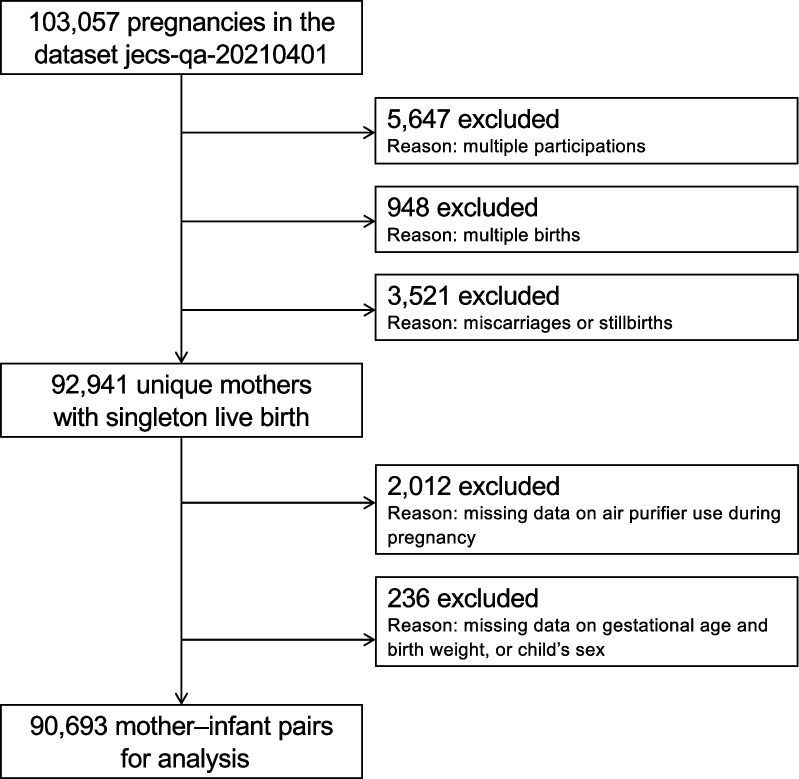
Flow chart of participation

The *jecs-qa-20210401* dataset, which was released in April 2021, was analyzed in this study. Among the 103,057 pregnancies in this dataset, 5647 were excluded due to multiple registrations, 948 due to multiple births, and 3521 following miscarriages or stillbirths. Among the remaining 92,941 unique mothers with live births, a further 2012 were excluded due to lack of response to the question on air purifier use during pregnancy and another 236 were excluded due to lack of response regarding gestational age, birth weight, or the child’s sex. Thus, the data from 90,693 mother–infant pairs were analyzed (Fig. 1).

### Measurements

A self-administered questionnaire was used to collect information from mothers during the second/third trimester on demographics, medical history, physical and mental health status, lifestyle factors, occupation, and socioeconomic status. Data from the medical records were transcribed by physicians, midwives/nurses, and/or research coordinators.

### Exposure

The use of air purifiers was assessed with a yes/no question “Have you used an air-cleaning device during the last year?” as described [20, 21].

### Outcomes

The primary outcome was the prevalence of ABOs, namely, PTB, SGA, and LBW, which were obtained from the medical records. PTB was defined as gestational age less than 37 weeks at delivery. SGA was defined as BW below the 10th percentile, accounting for parity, gestational age, and neonatal sex according to the Japan Pediatric Society guidelines [25]. Due to the lack of a gold-standard method for assessing SGA in infants born by cesarean section in Japan, only those born by vaginal delivery were analyzed in the SGA analysis, as described in our previous study [26]. BW was measured in grams and LBW was defined as BW less than 2500 g.

### Covariates

We used the following *a priori-*selected confounders: maternal age (< 25, 25-<30, 30-<35, or ≥ 35); body mass index (BMI; <18.5, 18.5-<25, or ≥ 25 kg/m^2^); parity (primipara or multipara); history of allergy (no or yes); Kessler Psychological Distress Scale [27–30] (K6; <5, 5–12, or ≥ 13); maternal smoking status (never, former, or current); maternal secondhand smoking status (almost never or never, once a week, 2–3 times a week, 4–6 times a week, or everyday); alcohol intake (never, former, or current); number of hours spent outdoors per day (< 1, 1-<2, 2-<3, or ≥ 3 per day); physical activity per day (no or yes); folic acid intake [31] (< 400 or ≥ 400 µg per day [32]); marital status (married, single, or divorced or widowed); highest education level (≤ 12, > 12-<16, or ≥ 16 years); employment status (unemployed, begun after pregnancy, quit after pregnancy, current); annual household income (≤ 4, 4-<6, or ≥ 6 million JPY); type of residence (wooden detached house, steel-frame detached house, wooden multiple dwelling house/apartment, steel-frame multiple dwelling house apartment, or other); number of rooms in the house/apartment (≤ 2, 3, 4, 5, or ≥ 6); living room flooring material (*tatami* [Japanese straw floor covering], carpet on *tatami*, wooden flooring/tile, carpet on wooden flooring/tile, or other); having a pet (no or yes); frequency of cleaning the living room floor with a vacuum cleaner (≤ 1–2 times a month, once a week, a few times a week, or everyday); frequency of cleaning futon bedding with a vacuum cleaner (almost never or never, a few times a year, 1–2 times a month, or ≥ once a week); frequency of airing the futon (almost never or never, a few times a year, 1–2 times a month, or ≥ once a week); using an anti-mite cover for the futon (no or yes); age of the house/apartment building (< 1, 1-<3, 3-<5, 5-<10, 10-<20, ≥ 20 years, or unknown); house renovation/interior finishing after becoming pregnant (yes or no); number of years living in current place of residence (< 1, 1-<3, 3-<5, 5-<10, 10-<20, or ≥ 20 years); and which of the 15 regional centers’ catchment areas they lived in. These covariates include standard variables for socioeconomic status and dwelling environment, and we selected several variables in terms of potential impact on exposure and/or outcome, as described previously [20, 21]. The categorization of these variables was performed in accordance with usual medical practice or common practice in Japan, and with reference to our previous studies [20, 21].

### Statistical analysis

To estimate the risk of ABOs according to air purifier use, we performed multivariable logistic regression analysis, which the 15 regional centers set as a random effect, to calculate the odds ratios (ORs) and their 95% confidence intervals (CIs). The exposure variable was the yes/no answer to the question regarding air purifier use, with non-use as the reference. Subgroup analysis stratified by infant sex was also performed.

Missing data were handled by multiple imputation. Missing response rates were 7.04% for annual household income, 4.03% for number of hours spent outdoors, 3.28% for physical activity, 3.05% for number of years living in current place of residence, 2.47% for parity, and ≤ 1% for all other covariates. Ten imputed datasets were created using chained equations [33], and standard rules were used to combine the results [34]. Data were analyzed using SAS ver. 9.4 software (SAS Institute Inc., Cary, NC).

### Additional analysis

Preterm birth and LBW often co-occur, so we have included LBW with full-term birth in an additional analysis. Furthermore, we performed an analysis with major congenital anomalies [35] as an outcome because they can also be considered adverse birth outcomes.

## Results

The characteristics of the mothers and dwelling environments together with data regarding air purifier use are shown in Table 1. Air purifier use was associated with parity, history of allergy, being married, high education level, high income, having a pet, and living in a newer house/apartment.

**Table 1:** Characteristics of the participants and dwelling environments

Variable	Category	Air purifier use			
		Yes		No	
		n	(%)	n	(%)
Subtotal		46,067	(50.8)	44,626	(49.2)
Age, y	< 25	4,273	(9.3)	5,251	(11.8)
	25–<30	13,420	(29.1)	12,274	(27.5)
	30–<35	16,817	(36.5)	15,044	(33.7)
	≥ 35	11,557	(25.1)	12,058	(27.0)
Body mass index, kg/m^2^	< 18.5	7,465	(16.2)	7,231	(16.2)
	18.5–<25	34,016	(73.8)	32,453	(72.7)
	≥ 25	4,586	(10.0)	4,943	(11.1)
Parity	Primipara	18,181	(39.5)	20,830	(46.7)
	Multipara	27,886	(60.5)	23,796	(53.3)
History of allergy	No	21,601	(46.9)	23,755	(53.2)
	Yes	24,466	(53.1)	20,871	(46.8)
Kessler Psychological Distress Scale score	< 5	32,825	(71.3)	31,470	(70.5)
	5–12	11,834	(25.7)	11,592	(26.0)
	≥ 13	1,408	(3.1)	1,564	(3.5)
Maternal smoking status	Never	26,544	(57.6)	25,848	(57.9)
	Former	17,640	(38.3)	16,508	(37.0)
	Current	1,883	(4.1)	2,270	(5.1)
Maternal secondhand smoking status	Almost never or never	28,949	(62.8)	27,255	(61.1)
	Once a week	5,652	(12.3)	5,198	(11.7)
	2–3 times a week	3,696	(8.0)	3,780	(8.5)
	4–6 times a week	2,150	(4.7)	2,316	(5.2)
	Everyday	5,619	(12.2)	6,077	(13.6)
Alcohol intake	Never	15,316	(33.3)	14,983	(33.6)
	Former	29,575	(64.2)	28,293	(63.4)
	Current	1,176	(2.6)	1,350	(3.0)
Number of hours spent outdoors per day	< 1	8,639	(18.8)	9,163	(20.5)
	1–<2	21,630	(47.0)	20,591	(46.1)
	2–<3	7,541	(16.4)	6,836	(15.3)
	≥ 3	8,257	(17.9)	8,036	(18.0)
Physical activity	No	10,240	(22.2)	10,742	(24.1)
	Yes	35,827	(77.8)	33,884	(75.9)
Folic acid intake per day, µg	< 400	40,419	(87.7)	39,564	(88.7)
	≥ 400	5,648	(12.3)	5,062	(11.3)
Marital status	Married	44,517	(96.6)	41,972	(94.1)
	Single	1,254	(2.7)	2,121	(4.8)
	Divorced or widowed	297	(0.6)	533	(1.2)
Highest education level, y	≤ 12	15,389	(33.4)	17,433	(39.1)
	> 12–<16	20,324	(44.1)	17,848	(40.0)
	≥ 16	10,354	(22.5)	9,345	(20.9)
Employment status	Unemployed	17,655	(38.3)	15,777	(35.4)
	Begun after pregnancy	743	(1.6)	672	(1.5)
	Quit after pregnancy	3,835	(8.3)	4,321	(9.7)
	Current	23,834	(51.7)	23,856	(53.5)
Annual household income, million yen	< 4	16,787	(36.4)	20,261	(45.4)
	4–<6	15,844	(34.4)	13,896	(31.1)
	≥ 6	13,436	(29.2)	10,470	(23.5)
Type of residence	Wooden detached house	19,225	(41.7)	18,087	(40.5)
	Steel-frame detached house	3,267	(7.1)	2,470	(5.5)
	Wooden multiple dwelling house/apartment	5,330	(11.6)	5,924	(13.3)
	Steel-frame multiple dwelling house/apartment	17,810	(38.7)	17,687	(39.6)
	Other	436	(1.0)	458	(1.0)
Number of rooms in house/apartment	≤ 2	7,723	(16.8)	9,374	(21.0)
	3	14,663	(31.8)	14,633	(32.8)
	4	9,485	(20.6)	7,832	(17.6)
	5	7,403	(16.1)	6,208	(13.9)
	≥ 6	6,794	(14.8)	6,579	(14.7)
Living room flooring material	*Tatami* (Japanese straw floor covering)	4,372	(9.5)	6,088	(13.6)
	Carpet on *tatami*	3,646	(7.9)	4,593	(10.3)
	Wooden flooring/tile	17,236	(37.4)	14,919	(33.4)
	Carpet on wooden flooring/tile	19,942	(43.3)	18,280	(41.0)
	Other	871	(1.9)	746	(1.7)
Having a pet	No	34,842	(75.6)	34,831	(78.1)
	Yes	11,225	(24.4)	9,796	(22.0)
Cleaning living room floor with vacuum cleaner	≤–2 times a month	3,128	(6.8)	4,419	(9.9)
	Once a week	13,466	(29.2)	14,460	(32.4)
	A few times a week	20,787	(45.1)	18,902	(42.4)
	Everyday	8,686	(18.9)	6,846	(15.3)
Cleaning futon with vacuum cleaner	Almost never or never	24,130	(52.4)	26,876	(60.2)
	A few times a year	6,048	(13.1)	5,334	(12.0)
	1–2 times a month	8,345	(18.1)	6,696	(15.0)
	≥ Once a week	7,545	(16.4)	5,720	(12.8)
Airing futon	Almost never or never	5,554	(12.1)	5,021	(11.3)
	A few times a year	8,400	(18.2)	8,743	(19.6)
	1–2 times a month	17,057	(37.0)	16,692	(37.4)
	≥ Once a week	15,055	(32.7)	14,171	(31.8)
Using anti-mite cover for futon or bedding after becoming pregnant	No	41,514	(90.1)	41,996	(94.1)
	Yes	4,554	(9.9)	2,630	(5.9)
Age of house/apartment building, y	< 1	2,973	(6.5)	2,121	(4.8)
	1–<3	5,986	(13.0)	4,134	(9.3)
	3–<5	4,781	(10.4)	3,772	(8.5)
	5–<10	7,516	(16.3)	6,476	(14.5)
	10–<20	10,519	(22.8)	10,591	(23.7)
	≥ 20	10,528	(22.9)	12,324	(27.6)
	Unknown	3,764	(8.2)	5,208	(11.7)
House renovation/interior finishing after becoming pregnant	Yes	1,602	(3.5)	1,270	(2.9)
	No	44,466	(96.5)	43,356	(97.2)
Number of years living in current place of residence	< 1	3,495	(7.6)	3,428	(7.7)
	1–<3	19,685	(42.7)	18,490	(41.4)
	3–<5	10,755	(23.4)	9,508	(21.3)
	5–<10	8,205	(17.8)	8,267	(18.5)
	10–<20	2,344	(5.1)	2,835	(6.4)
	≥ 20	1,583	(3.4)	2,099	(4.7)
Area of residence	A	3,780	(8.2)	3,466	(7.8)
	B	3,710	(8.1)	4,426	(9.9)
	C	6,346	(13.8)	5,509	(12.3)
	D	2,768	(6.0)	2,306	(5.2)
	E	3,265	(7.1)	2,645	(5.9)
	F	2,884	(6.3)	3,636	(8.2)
	G	2,391	(5.2)	2,593	(5.8)
	H	2,659	(5.8)	2,381	(5.3)
	I	1,796	(3.9)	1,613	(3.6)
	J	4,044	(8.8)	3,045	(6.8)
	K	2,527	(5.5)	2,045	(4.6)
	L	1,191	(2.6)	1,501	(3.4)
	M	2,900	(6.3)	3,356	(7.5)
	N	3,690	(8.0)	3,114	(7.0)
	O	2,116	(4.6)	2,990	(6.7)

The prevalence of primary outcomes was 4.5% for PTB, 7.4% for SGA, and 8.1% for LBW. The case, prevalence, crude ORs, and adjusted ORs for each ABO according to air purifier use or non-use are shown in Table 2. The crude model analysis revealed that the ORs (95% CIs) for PTB, SGA, and LBW in the group that used an air purifier were 0.92 (0.87, 0.98, *p* = 0.014), 0.94 (0.89, 1.00, *p* = 0.033), and 0.89 (0.85, 0.93, *p* < 0.001), respectively. The association disappeared in the adjusted model for PTB (0.96 [0.90, 1.02], *p* = 0.223) but remained significant for SGA (0.94 [0.89, 1.00], *p* = 0.048) and LBW (0.93 [0.88, 0.98], *p* = 0.003). Subgroup analysis stratified by infant sex revealed significantly lower ORs for LBW in both models for male infants (crude and adjusted ORs [95% CIs] 0.87 [0.81, 0.94, *p* < 0.001] and 0.91 [0.85, 0.98, *p* = 0.017], respectively) but in only the crude model for female infants (0.90 [0.85, 0.96, *p* = 0.002] and 0.94 [0.88, 1.00, *p* = 0.055], respectively) (Table 3).

**Table 2:** Cases, prevalences, and crude and adjusted OR (95% CIs) according to use or non-use of air purifiers

	Air purifier		
	Use	Non-use	*p*
PTB			
Cases, *n*	2,010	2,099	
Subtotal, *n*	46,067	44,626	
Prevalence, %	4.36	4.70	
Crude OR	0.92 (0.87, 0.98)	1.00 (Ref.)	0.014
Adjusted OR^a^	0.96 (0.90, 1.02)	1.00 (Ref.)	0.223
SGA			
Cases, *n*	2,682	2,738	
Subtotal, *n*	37,382	36,081	
Prevalence, %	7.17	7.59	
Crude OR	0.94 (0.89, 1.00^b^)	1.00 (Ref.)	0.033
Adjusted OR^a^	0.94 (0.89, 1.00^c^)	1.00 (Ref.)	0.048
LBW			
Cases, *n*	3,522	3,798	
Subtotal, *n*	46,067	44,626	
Prevalence, %	7.65	8.51	
Crude OR	0.89 (0.85, 0.93)	1.00 (Ref.)	< 0.001
Adjusted OR^a^	0.93 (0.88, 0.98)	1.00 (Ref.)	0.003

*PTB *Preterm birth, *SGA *Small for gestational age, *LBW L*ow birth weight, *OR *Odds ratio, *CI *Confidence interval, *Ref *Reference

^a^Adjusted for all variables shown in Table 1

^b^More precisely, 0.995

^c^More precisely, 0.9995

**Table 3:** Cases, prevalences, and crude and adjusted OR (95% CIs) according to use or non-use of air purifiers by infant sex

Infant sex	Male			Female		
	Air purifier			Air purifier		
	Use	Non-use	*p*	Use	Non-use	*p*
PTB						
Cases, *n*	1,149	1,241		861	858	
Subtotal, *n*	23,500	23,003		22,567	21,623	
Prevalence, %	4.89	5.39		3.82	3.97	
Crude OR	0.90 (0.83, 0.98)	1.00 (Ref.)	0.014	0.96 (0.87, 1.06)	1.00 (Ref.)	0.407
Adjusted OR^a^	0.93 (0.86, 1.02)	1.00 (Ref.)	0.109	1.00 (0.91, 1.11)	1.00 (Ref.)	0.979
SGA						
Cases, *n*	1,352	1,391		1,330	1,347	
Subtotal, *n*	19,035	18,611		18,347	17,470	
Prevalence, %	7.10	7.48		7.25	7.71	
Crude OR	0.95 (0.88, 1.02)	1.00 (Ref.)	0.169	0.94 (0.86, 1.01)	1.00 (Ref.)	0.098
Adjusted OR^a^	0.95 (0.88, 1.03)	1.00 (Ref.)	0.214	0.94 (0.87, 1.03)	1.00 (Ref.)	0.142
LBW						
Cases, *n*	1,568	1,744		1,954	2,053	
Subtotal, *n*	23,500	23,003		22,567	21,623	
Prevalence, %	6.67	7.58		8.66	9.50	
Crude OR	0.87 (0.81, 0.94)	1.00 (Ref.)	< 0.001	0.90 (0.85, 0.96)	1.00 (Ref.)	0.002
Adjusted OR^a^	0.91 (0.85, 0.98)	1.00 (Ref.)	0.017	0.94 (0.88, 1.00^b^)	1.00 (Ref.)	0.055

*PTB* Preterm birth, *SGA* Small for gestational age, *LBW* Low birth weight, *OR* Odds ratio, *CI* Confidence interval, *Ref* Reference

^a^Adjusted for all variables shown in Table 1

^b^More precisely, 1.002

The results of the additional analyses—LBW focused on full-term births and major congenital anomalies—are summarized in Supplemental Table 1. The adjusted OR (95% CI) for LBW remained significant (0.92 [0.87, 0.98]) when the analysis was restricted to full-term births.

## Discussion

Accumulating evidence indicates that prenatal exposure to air pollutants has harmful impacts on pregnancy and birth outcomes [5, 10, 11, 36–40]. Identifying strategies for preventing ABOs that are highly relevant and cost-effective is crucial from a public health standpoint. In this study, we found that air purifier use during pregnancy was associated with a reduced prevalence of SGA and LBW compared with non-use. Thus, implementation of air quality management and other efforts to reduce air pollution may reduce the prevalence of ABOs among pregnant populations. Other methods that reduce air pollution may produce a similar effect, such as closing windows to keep out polluted air, ensuring appropriate ventilation at home, and wearing a mask.

Air pollutants induce inflammation and oxidative stress, which are thought to be the main mechanisms underlying ABOs [41, 42]. One typical air pollutant is PM2.5. A cell culture study showed that PM2.5 can enter trophoblasts and induce mitochondrial vacuolization and dysfunction as well as endoplasmic reticulum stress [43]. Moreover, production of the inflammatory mediator interleukin-6 was increased and production of human chorionic gonadotropin was decreased after the first trimester trophoblast cell line (HTR-8/SVneo) was exposed to PM2.5 [44]. Janssen et al. reported that exposure to PM2.5 was associated with changes in fetal thyroid hormone levels, which may contribute to reduced BW [45]. Their study also suggested that PM2.5 exposure during the third trimester in particular might increase the risk of LBW [45]. These mechanisms may lead to abnormal fetal development and to abnormal fetal weight gain. Thus, air purifier use during pregnancy that effectively reduces the concentrations of air pollutants in indoor air may be attributable to the favorable impact on LBW.

The developing fetus has inadequate antioxidant defense systems and is thus susceptible to oxidative damage induced by environmental pollutants. Females typically exhibit stronger innate and adaptive immune responses compared with males *in utero* [46]. Previous studies have reported that female newborns are more susceptible than male newborns to LBW due to preconception or prenatal air pollution exposure [16, 47]. One study reported that female fetuses have lower antioxidant responses compared with male fetuses [48]. However, another study indicated that female fetuses appear to be less susceptible to oxidative stress [49]. The placenta from premature male fetuses tends to be more inflamed compared with those from female fetuses, suggesting that a maternal immune reaction to fetal tissue may be more common in male fetuses [50]. It is also possible that air pollutants capable of inducing oxidative stress may induce different responses according to the sex of the fetus. However, the differences in antioxidant pathways between male and female fetuses following intrauterine exposure to environmental pollutants is not completely understood. Therefore, further study is required to clarify the precise factors mediating these sex differences in responses, which likely reflect the complex interactions between sex and environment *in utero*.

Our results suggest that the favorable effect of air purifier use was more pronounced on SGA and LBW compared with PTB. Although the exact reasons for this difference need to be clarified in future studies, several mechanisms are suggested. One possibility is that the three ABOs have different etiologies, each of which is affected by air pollutants in different ways. BW can be influenced by individual and environmental factors [4]. PTB is thought to be a syndrome caused by multiple mechanisms, including inflammation, uteroplacental ischemia or hemorrhage, stress, and other immunologically mediated processes [51]. PTB can be initiated by cervical softening and ripening, myometrial contractions, and decidual membrane activation [51]. Meanwhile, SGA may be triggered by an abnormal reaction between trophoblasts and uterine tissues in the first few weeks of pregnancy [52, 53]. SGA is also determined by BW and gestational age at delivery. These different mechanisms and the susceptibility to air pollutants of each ABO might explain the different effects of air purifier use. A previous study reported that LBW was more consistently correlated with maternal exposure to PM2.5 compared with PTB [36]. Incidentally, SGA is not significantly different when the analysis is stratified by sex, but this can be attributed to a decrease in statistical power simply because the number of analyses is almost halved by stratification, given that the OR itself is almost the same in the overall and stratified analyses.

Another possibility is that each ABO has a different susceptibility window. Previous studies have indicated that air pollution exposure in the earlier stages of pregnancy may impact the embryo and the formation of the placenta, leading to PTB or SGA, whereas exposure in late pregnancy may effect BW, given that this period is critical for fetal weight gain [5, 37, 47, 54–57]. Thus, air purifier use in different trimesters may affect the prevalence of different ABOs. We examined air purifier use with a simple yes/no question and could therefore not evaluate the impact of air purifier use by trimester.

The main strengths of this study include that it was a large prospective cohort study with a high response rate under a national birth cohort [24]. The prevalence of PTB and that of LBW derived from this study were well consistent with the results from the Japanese Vital Statistics [24], which suggests that this study is highly representative of the general Japanese population. In addition, the prospective collection of exposure and outcome data minimized recall bias, and important confounders were included in the model.

We also recognize some limitations of the study. As mentioned above, we evaluated the air purifier use with a simple yes/no question. Further analyses of the performance of various air purifiers, filter types, and the frequency and duration of use should also be considered. Because the data were obtained using a questionnaire, self-reported biases may be present. Moreover, we did not measure indoor concentrations of particulate matter or other air pollutants, which did not allow for mediation analysis using the concentrations of air pollutants. Although paternal exposure to environmental pollution is also reported to influence BW [58], we could not obtain this information for fathers. Due to the observational nature of the study, the results may have been confounded by unpredicted or poorly measured potential residual factors.

## Conclusions

We found a negative association between air purifier use during pregnancy and the prevalence of SGA and LBW. Our results suggest that using an air purifier or other methods to reduce air pollution may be beneficial for reducing the prevalence of SGA and LBW. Implementation of air quality management to reduce air pollutants during pregnancy may lead to lower prevalence of SGA and LBW or other ABOs and associated disease burden.

## Supplementary Information

Supplementary Material 1.

## Data Availability

Data are unsuitable for public deposition due to ethical restrictions and legal framework of Japan. It is prohibited by the Act on the Protection of Personal Information (Act No. 57 of 30 May 2003, amendment on 9 September 2015) to publicly deposit the data containing personal information. Ethical Guidelines for Medical and Health Research Involving Human Subjects enforced by the Japan Ministry of Education, Culture, Sports, Science and Technology and the Ministry of Health, Labour and Welfare also restricts the open sharing of the epidemiologic data. All inquiries about access to data should be sent to: jecs-en@nies.go.jp. The person responsible for handling enquiries sent to this e-mail address is Dr Shoji F. Nakayama, JECS Programme Office, National Institute for Environmental Studies.

## Abbreviations

ABO: Adverse birth outcome
BMI: Body mass index
BW: Birth weight
CI: Confidence interval
JECS: Japan Environment and Children’s Study
JPY: Japanese Yen
LBW: Low birth weight
OR: Odds ratio
PTB: Preterm birth
SGA: Small for gestational age

## References

[1] Morken NH, Kallen K, Jacobsson B. Outcomes of preterm children according to type of delivery onset: a nationwide population-based study. Paediatr Perinat Epidemiol. 2007;21(5):458–64. 17697076 10.1111/j.1365-3016.2007.00823.x

[2] Lindstrom K, Winbladh B, Haglund B, Hjern A. Preterm infants as young adults: a Swedish national cohort study. Pediatrics. 2007;120(1):70–7. 17606563 10.1542/peds.2006-3260

[3] Ray JG, Park AL, Fell DB. Mortality in infants affected by preterm birth and severe small-for-gestational age birth weight. Pediatrics. 2017;140(6):e20171881. 29117948 10.1542/peds.2017-1881

[4] Inadera H. Developmental origins of obesity and type 2 diabetes: molecular aspects and role of chemicals. Environ Health Prev Med. 2013;18(3):185–97. 23382021 10.1007/s12199-013-0328-8 PMC3650171

[5] Sun X, Luo X, Zhao C, Zhang B, Tao J, Yang Z, et al. The associations between birth weight and exposure to fine particulate matter (PM2.5) and its chemical constituents during pregnancy: a meta-analysis. Environ Pollut. 2016;211:38–47. 26736054 10.1016/j.envpol.2015.12.022

[6] van den Hooven EH, de Kluizenaar Y, Pierik FH, Hofman A, van Ratingen SW, Zandveld PY, et al. Chronic air pollution exposure during pregnancy and maternal and fetal C-reactive protein levels: the Generation R Study. Environ Health Perspect. 2012;120(5):746–51. 22306530 10.1289/ehp.1104345 PMC3346784

[7] Varshavsky J, Smith A, Wang A, Hom E, Izano M, Huang H, et al. Heightened susceptibility: a review of how pregnancy and chemical exposures influence maternal health. Reprod Toxicol. 2020;92:14–56. 31055053 10.1016/j.reprotox.2019.04.004 PMC6824944

[8] Hettfleisch K, Carvalho MA, Hoshida MS, Pastro LDM, Saldiva S, Vieira SE, et al. Individual exposure to urban air pollution and its correlation with placental angiogenic markers in the first trimester of pregnancy, in Sao Paulo, Brazil. Environ Sci Pollut Res Int. 2021;28(22):28658–65. 33544347 10.1007/s11356-021-12353-7

[9] Zhang S, Mwiberi S, Pickford R, Breitner S, Huth C, Koenig W, et al. Longitudinal associations between ambient air pollution and insulin sensitivity: results from the KORA cohort study. Lancet Planet Health. 2021;5(1):e39-49. 33421408 10.1016/S2542-5196(20)30275-8

[10] Stieb DM, Chen L, Eshoul M, Judek S. Ambient air pollution, birth weight and preterm birth: a systematic review and meta-analysis. Environ Res. 2012;117:100–11. 22726801 10.1016/j.envres.2012.05.007

[11] Ghosh R, Causey K, Burkart K, Wozniak S, Cohen A, Brauer M. Ambient and household PM2.5 pollution and adverse perinatal outcomes: a meta-regression and analysis of attributable global burden for 204 countries and territories. PLoS Med. 2021;18(9):e1003718. 34582444 10.1371/journal.pmed.1003718 PMC8478226

[12] Rich DQ, Liu K, Zhang J, Thurston SW, Stevens TP, Pan Y, et al. Differences in Birth Weight Associated with the 2008 Beijing Olympics Air Pollution reduction: results from a natural experiment. Environ Health Perspect. 2015;123(9):880–7. 25919693 10.1289/ehp.1408795 PMC4559955

[13] Parker JD, Mendola P, Woodruff TJ. Preterm birth after the Utah Valley Steel Mill closure: a natural experiment. Epidemiology. 2008;19(6):820–3. 18854706 10.1097/EDE.0b013e3181883d5d

[14] Ministry of Health, Labour and Welfare: The 8th study group on supporting the balance between work and Child/Family care: appendix 3. 2023. https://www.mhlw.go.jp/stf/wp/hakusyo/roudou/23/23-1.html. Accessed 20 Sep 2024 (in Japanese).

[15] Pope DP, Mishra V, Thompson L, Siddiqui AR, Rehfuess EA, Weber M, et al. Risk of low birth weight and stillbirth associated with indoor air pollution from solid fuel use in developing countries. Epidemiol Rev. 2010;32:70–81. 20378629 10.1093/epirev/mxq005

[16] Lu C, Deng M, Norbäck D, Liu Z, Murithi R, Gakii., Deng Q. Effect of outdoor air pollution and indoor environmental factors on small for gestational age. Build Environ. 2021;206:108399.

[17] Zhang S, Hu H, Liu X, Liu Z, Mao Y, Li Z, et al. The impact of household fuel usage on adverse pregnancy outcomes in rural Ma’anshan City, Anhui Province: a birth cohort study. Environ Sci Pollut Res Int. 2023;30(45):100950–8. 37644269 10.1007/s11356-023-29543-0

[18] Kanatani KT, Okumura M, Tohno S, Adachi Y, Sato K, Nakayama T. Indoor particle counts during Asian dust events under everyday conditions at an apartment in Japan. Environ Health Prev Med. 2014;19(1):81–8. 23934359 10.1007/s12199-013-0356-4 PMC3890080

[19] Fermo P, Artinano B, De Gennaro G, Pantaleo AM, Parente A, Battaglia F, et al. Improving indoor air quality through an air purifier able to reduce aerosol particulate matter (PM) and volatile organic compounds (VOCs): experimental results. Environ Res. 2021;197:111131. 33865819 10.1016/j.envres.2021.111131

[20] Matsumura K, Hamazaki K, Tsuchida A, Inadera H, JECS Group. Prospective association of air-purifier usage during pregnancy with infant neurodevelopment: a nationwide longitudinal study – Japan Environment and Children’s study (JECS). J Clin Med. 2020;9(6):1924. 32575520 10.3390/jcm9061924 PMC7356334

[21] Matsumura K, Hamazaki K, Tsuchida A, Inadera H, JECS Group. Prospective association of air purifier use during pregnancy with the neurodevelopment of toddlers in the Japan Environment and Children’s study. Sci Rep. 2021;11(1):19454. 34593840 10.1038/s41598-021-98482-y PMC8484572

[22] Iwai-Shimada M, Nakayama SF, Isobe T, Michikawa T, Yamazaki S, Nitta H, et al. Questionnaire results on exposure characteristics of pregnant women participating in the Japan Environment and Children Study (JECS). Environ Health Prev Med. 2018;23(1):45. 30219031 10.1186/s12199-018-0733-0 PMC6138908

[23] Kawamoto T, Nitta H, Murata K, Toda E, Tsukamoto N, Hasegawa M, et al. Rationale and study design of the Japan environment and children’s study (JECS). BMC Public Health. 2014;14:25. 24410977 10.1186/1471-2458-14-25 PMC3893509

[24] Michikawa T, Nitta H, Nakayama SF, Yamazaki S, Isobe T, Tamura K, et al. Baseline profile of participants in the Japan Environment and Children’s study (JECS). J Epidemiol. 2018;28(2):99–104. 29093304 10.2188/jea.JE20170018 PMC5792233

[25] Itabashi K, Fujimura M, Kusuda S. Introduction of the new standard for birth size by gestational ages. J Jpn Pediatr Soc. 2010;114:1271–93 (in Japanese).

[26] Inadera H, Takamori A, Matsumura K, Tsuchida A, Cui ZG, Hamazaki K, et al. Association of blood cadmium levels in pregnant women with infant birth size and small for gestational age infants: the Japan Environment and Children’s study. Environ Res. 2020;191:110007. 32768474 10.1016/j.envres.2020.110007

[27] Furukawa TA, Kawakami N, Saitoh M, Ono Y, Nakane Y, Nakamura Y, et al. The performance of the Japanese version of the K6 and K10 in the World Mental Health Survey Japan. Int J Methods Psychiatr Res. 2008;17(3):152–8. 18763695 10.1002/mpr.257 PMC6878390

[28] Kessler RC, Andrews G, Colpe LJ, Hiripi E, Mroczek DK, Normand SL, et al. Short screening scales to monitor population prevalences and trends in non-specific psychological distress. Psychol Med. 2002;32(6):959–76. 12214795 10.1017/s0033291702006074

[29] Sakurai K, Nishi A, Kondo K, Yanagida K, Kawakami N. Screening performance of K6/K10 and other screening instruments for mood and anxiety disorders in Japan. Psychiatry Clin Neurosci. 2011;65(5):434–41. 21851452 10.1111/j.1440-1819.2011.02236.x

[30] Matsumura K, Hamazaki K, Tsuchida A, Inadera H, JECS Group. Pet ownership during pregnancy and mothers’ mental health conditions up to 1 year postpartum: a nationwide birth cohort-the Japan Environment and Children’s study. Soc Sci Med. 2022;309:115216. 36029711 10.1016/j.socscimed.2022.115216

[31] Yokoyama Y, Takachi R, Ishihara J, Ishii Y, Sasazuki S, Sawada N, et al. Validity of short and long self-administered food frequency questionnaires in ranking Dietary Intake in Middle-aged and Elderly Japanese in the Japan Public Health Center-based prospective study for the Next Generation (JPHC-NEXT) Protocol Area. J Epidemiol. 2016;26(8):420–32. 27064130 10.2188/jea.JE20150064 PMC4967663

[32] Nishigori H, Nishigori T, Obara T, Suzuki T, Mori M, Imaizumi K, et al. Prenatal folic acid supplement/dietary folate and cognitive development in 4-year-old offspring from the Japan Environment and Children’s study. Sci Rep. 2023;13(1):9541. 37308528 10.1038/s41598-023-36484-8 PMC10260997

[33] van Buuren S. Multiple imputation of discrete and continuous data by fully conditional specification. Stat Methods Med Res. 2007;16(3):219–42. 17621469 10.1177/0962280206074463

[34] Rubin DB. Multiple imputation for nonresponse in surveys. New York: Wiley; 2004.

[35] Mezawa H, Tomotaki A, Yamamoto-Hanada K, Ishitsuka K, Ayabe T, Konishi M, et al. Prevalence of congenital anomalies in the Japan Environment and Children’s study. J Epidemiol. 2019;29(7):247–56. 30249945 10.2188/jea.JE20180014 PMC6556438

[36] Lavigne E, Yasseen AS 3rd, Stieb DM, Hystad P, van Donkelaar A, Martin RV, et al. Ambient air pollution and adverse birth outcomes: differences by maternal comorbidities. Environ Res. 2016;148:457–66. 27136671 10.1016/j.envres.2016.04.026

[37] Yuan L, Zhang Y, Gao Y, Tian Y. Maternal fine particulate matter (PM(2.5)) exposure and adverse birth outcomes: an updated systematic review based on cohort studies. Environ Sci Pollut Res Int. 2019;26(14):13963–83. 30891704 10.1007/s11356-019-04644-x

[38] Wang W, Mu S, Yan W, Ke N, Cheng H, Ding R. Prenatal PM2.5 exposure increases the risk of adverse pregnancy outcomes: evidence from meta-analysis of cohort studies. Environ Sci Pollut Res Int. 2023;30(48):106145–97. 37723397 10.1007/s11356-023-29700-5

[39] Zhang Y, Ye T, Yu P, Xu R, Chen G, Yu W, et al. Preterm birth and term low birth weight associated with wildfire-specific PM(2.5): a cohort study in New South Wales, Australia during 2016–2019. Environ Int. 2023;174:107879. 36958111 10.1016/j.envint.2023.107879

[40] Song S, Gao Z, Zhang X, Zhao X, Chang H, Zhang J, et al. Ambient fine particulate matter and pregnancy outcomes: an umbrella review. Environ Res. 2023;235:116652. 37451569 10.1016/j.envres.2023.116652

[41] Ferguson KK, McElrath TF, Pace GG, Weller D, Zeng L, Pennathur S, et al. Urinary polycyclic aromatic Hydrocarbon Metabolite associations with biomarkers of inflammation, angiogenesis, and oxidative stress in pregnant women. Environ Sci Technol. 2017;51(8):4652–60. 28306249 10.1021/acs.est.7b01252 PMC5771235

[42] Heydari H, Najafi ML, Akbari A, Rezaei H, Miri M. Prenatal exposure to traffic-related air pollution and glucose homeostasis: a cross-sectional study. Environ Res. 2021;201:111504. 34144009 10.1016/j.envres.2021.111504

[43] Familari M, Naav A, Erlandsson L, de Iongh RU, Isaxon C, Strandberg B, et al. Exposure of trophoblast cells to fine particulate matter air pollution leads to growth inhibition, inflammation and ER stress. PLoS ONE. 2019;14(7):e0218799. 31318865 10.1371/journal.pone.0218799 PMC6638881

[44] Naav A, Erlandsson L, Isaxon C, Asander Frostner E, Ehinger J, Sporre MK, et al. Urban PM2.5 induces Cellular toxicity, hormone dysregulation, oxidative damage, inflammation, and mitochondrial interference in the HRT8 trophoblast cell line. Front Endocrinol (Lausanne). 2020;11:75. 32226408 10.3389/fendo.2020.00075 PMC7080655

[45] Janssen BG, Saenen ND, Roels HA, Madhloum N, Gyselaers W, Lefebvre W, et al. Fetal thyroid function, Birth Weight, and in Utero exposure to fine particle Air Pollution: a birth cohort study. Environ Health Perspect. 2017;125(4):699–705. 27623605 10.1289/EHP508 PMC5382000

[46] Klein SL, Flanagan KL. Sex differences in immune responses. Nat Rev Immunol. 2016;16(10):626–38. 27546235 10.1038/nri.2016.90

[47] Liao J, Zhang Y, Yang Z, Qiu C, Chen W, Zhang JJ, et al. Identifying critical windows of air pollution exposure during preconception and gestational period on birthweight: a prospective cohort study. Environ Health. 2023;22(1):71. 37858139 10.1186/s12940-023-01022-6 PMC10585741

[48] Al-Gubory KH. Multiple exposures to environmental pollutants and oxidative stress: is there a sex specific risk of developmental complications for fetuses? Birth Defects Res C Embryo Today. 2016;108(4):351–64. 28033658 10.1002/bdrc.21142

[49] Kander MC, Cui Y, Liu Z. Gender difference in oxidative stress: a new look at the mechanisms for cardiovascular diseases. J Cell Mol Med. 2017;21(5):1024–32. 27957792 10.1111/jcmm.13038 PMC5387169

[50] Goldenberg RL, Andrews WW, Faye-Petersen OM, Goepfert AR, Cliver SP, Hauth JC. The Alabama Preterm Birth Study: intrauterine infection and placental histologic findings in preterm births of males and females less than 32 weeks. Am J Obstet Gynecol. 2006;195(6):1533–7. 16796981 10.1016/j.ajog.2006.05.023

[51] Goldenberg RL, Culhane JF, Iams JD, Romero R. Epidemiology and causes of preterm birth. Lancet. 2008;371(9606):75–84. 18177778 10.1016/S0140-6736(08)60074-4 PMC7134569

[52] Chauhan SP, Rice MM, Grobman WA, Bailit J, Reddy UM, Wapner RJ, et al. Neonatal morbidity of small- and large-for-gestational-age neonates born at term in uncomplicated pregnancies. Obstet Gynecol. 2017;130(3):511–9. 28796674 10.1097/AOG.0000000000002199 PMC5578445

[53] Le HQ, Batterman SA, Wirth JJ, Wahl RL, Hoggatt KJ, Sadeghnejad A, et al. Air Pollutant exposure and preterm and term small-for-gestational-age births in Detroit, Michigan: long-term trends and associations. Environ Int. 2012;44:7–17. 22314199 10.1016/j.envint.2012.01.003 PMC4331339

[54] Hsieh TT, Hsu JJ, Chen CJ, Chiu TH, Liou JD, Hsieh CC, et al. Analysis of birth weight and gestational age in Taiwan. J Formos Med Assoc. 1991;90(4):382–7. 1680968

[55] Selevan SG, Kimmel CA, Mendola P. Identifying critical windows of exposure for children’s health. Environ Health Perspect. 2000;108(Suppl 3):451–5. 10852844 10.1289/ehp.00108s3451 PMC1637810

[56] Yuan L, Zhang Y, Wang W, Chen R, Liu Y, Liu C, et al. Critical windows for maternal fine particulate matter exposure and adverse birth outcomes: the Shanghai birth cohort study. Chemosphere. 2020;240:124904. 31550593 10.1016/j.chemosphere.2019.124904

[57] Liu J, Chen Y, Liu D, Ye F, Sun Q, Huang Q, et al. Prenatal exposure to particulate matter and term low birth weight: systematic review and meta-analysis. Environ Sci Pollut Res Int. 2023;30(23):63335–46. 37059952 10.1007/s11356-023-26831-7 PMC10172254

[58] Robledo CA, Yeung E, Mendola P, Sundaram R, Maisog J, Sweeney AM, et al. Preconception maternal and paternal exposure to persistent organic pollutants and birth size: the LIFE study. Environ Health Perspect. 2015;123(1):88–94. 25095280 10.1289/ehp.1308016 PMC4286275

