# Supplementary material for: Association of air purifier usage during pregnancy with adverse birth outcomes: the Japan Environment and Children’s Study
Source: BMC Public Health. 2024 Dec 18;24:3421. doi: 10.1186/s12889-024-20802-4 (PMC11654371; doi:10.1186/s12889-024-20802-4)
Supplement: Supplementary file 1 — Supplementary Material 1. [file 12889_2024_20802_MOESM1_ESM.docx]

**Supplementary Table 1**. Cases, prevalences, and crude and adjusted OR (95% CIs) according to use or non-use of air purifiers.

|  |  | Air purifier |  |
| --- | --- | --- | --- |
|  |  | Use | Non-use |
| LBW only for full-term birth | | |  |
|  | Cases, *n* | 2,287 | 2,473 |
|  | Subtotal, *n* | 43,938 | 42,428 |
|  | Prevalence, % | 5.20 | 5.83 |
|  | Crude OR | 0.89 (0.84, 0.94) | 1.00 (Ref.) |
|  | Adjusted OR^a^ | 0.92 (0.87, 0.98) | 1.00 (Ref.) |
| Major congenital anomaly | | |  |
|  | Cases, *n* | 1,067 | 1,090 |
|  | Subtotal, *n* | 46,067 | 44,626 |
|  | Prevalence, % | 2.32 | 2.44 |
|  | Crude OR | 0.95 (0.87, 1.03) | 1.00 (Ref.) |
|  | Adjusted OR^a^ | 0.97 (0.88, 1.06) | 1.00 (Ref.) |

^a^Adjusted for all variables shown in Table 1.

PTB, preterm birth; SGA, small for gestational age; LBW, low birth weight; OR, odds ratio; CI, confidence interval; Ref, reference
